# Future Directions and Evolution Strategies for the Clinical Management of Traumatic Brain Injury

**DOI:** 10.3390/jcm15166236

**Published:** 2026-08-12

**Authors:** Adam J. Wells, Abhiram D. Hiwase, Ivan Timofeev, Peter J. A. Hutchinson

**Affiliations:** 1 Department of Neurosurgery, Royal Adelaide Hospital, Adelaide, SA 5000, Australia; 2 School of Medicine, College of Health, Adelaide University, Adelaide, SA 5000, Australia; 3 Division of Neurosurgery, Department of Clinical Neurosciences, Cambridge University Hospital NHS Foundation Trust, Cambridge CB2 0QQ, UK

**Keywords:** traumatic brain injury, clinical management, neurocritical care, neuromonitoring, neuroprotection, artificial intelligence, machine learning

## Abstract

The management of traumatic brain injury remains one of the greatest challenges in modern medicine. Determining whom to treat, when to intervene, and how aggressively to proceed and persist continues to define the central dilemma of neurotrauma care. While the injured brain possesses only limited regenerative capacity, substantial opportunity remains to mitigate the cascade of secondary brain injury. Previous technological and scientific advances have progressively challenged the therapeutic nihilism historically associated with severe neurotrauma, and the accelerating pace of innovation is invigorating efforts to push the boundaries of care. Contemporary neurotrauma research increasingly seeks to reduce the incidence of injury through prevention, enable ultra-early diagnosis and intervention, biologically subclassify heterogeneous injury patterns, and integrate multimodal neuromonitoring into precision-guided therapeutic strategies. Advances in artificial intelligence, computational modelling, imaging, biomarker science, and neurocritical care now build upon decades of foundational work established by prior generations of clinicians and scientists. Together, these developments may enable increasingly individualised approaches to neurotrauma management, with the aim of preserving meaningful neurological function, quality of life, and human potential on a global scale. Although many challenges remain, modern neurotrauma increasingly stands at the threshold of such transformation. This narrative review explores the emerging technologies, evolving paradigms, and future directions that may shape the next era of neurotrauma care.

## 1. Introduction

Despite modern advances in the clinical management of traumatic brain injury (TBI), the global burden of this disease remains devastating, and TBI is still the leading worldwide cause of traumatic death and disability. It is currently estimated that up to 69 million people each year sustain a TBI [1], and the chronicity of the condition, coupled with the associated disability and decreased productivity, results in TBI indirectly consuming up to 0.5% of global economic activity [2]. The numbers are staggering, yet TBI is still considered the “silent epidemic”, with profound impacts on survivors, their families, the wider community and society [3]. Resource consumption and lost productivity contribute significantly to global burden and published figures are almost certainly underreported, which further highlights the extent of the problem. TBI management strategies need urgent attention to greatly reduce mortality, limit disability, and reverse the economic burden, and a paradigm shift to facilitate measures towards significantly improving TBI outcomes in the digital age is desperately required.

The last major paradigm shift in TBI management took place half a century ago. “Assessment of Coma and Impaired Consciousness” was published in *The Lancet* in 1974, and the simultaneous explosion of neuroscience research, the development of modern neurocritical care, and the acceptance of Teasdale and Jennett’s Glasgow Coma Scale (GCS) scoring system in the 1970s helped usher in the current golden age of managing TBI [4]. The last 50 years has seen: the rapid development of advanced neuroradiological investigations that aid TBI diagnosis and help direct management; sophisticated neuromonitoring technologies, including not only intracranial pressure measurement but also tissue oxygenation, microdialysis and cerebral compliance; protocol driven surgical and neurocritical care interventions, including evidence regarding decompressive craniectomy and the optimal timing of cranioplasty; and an increased understanding of the pathophysiological mechanisms that affect arguably the most complex organ in the human body. Artificial intelligence (AI) is poised to produce the next evolution in healthcare, and the next 50 years are likely to bring similarly transformative change to the clinical management of TBI.

**Figure 1:**
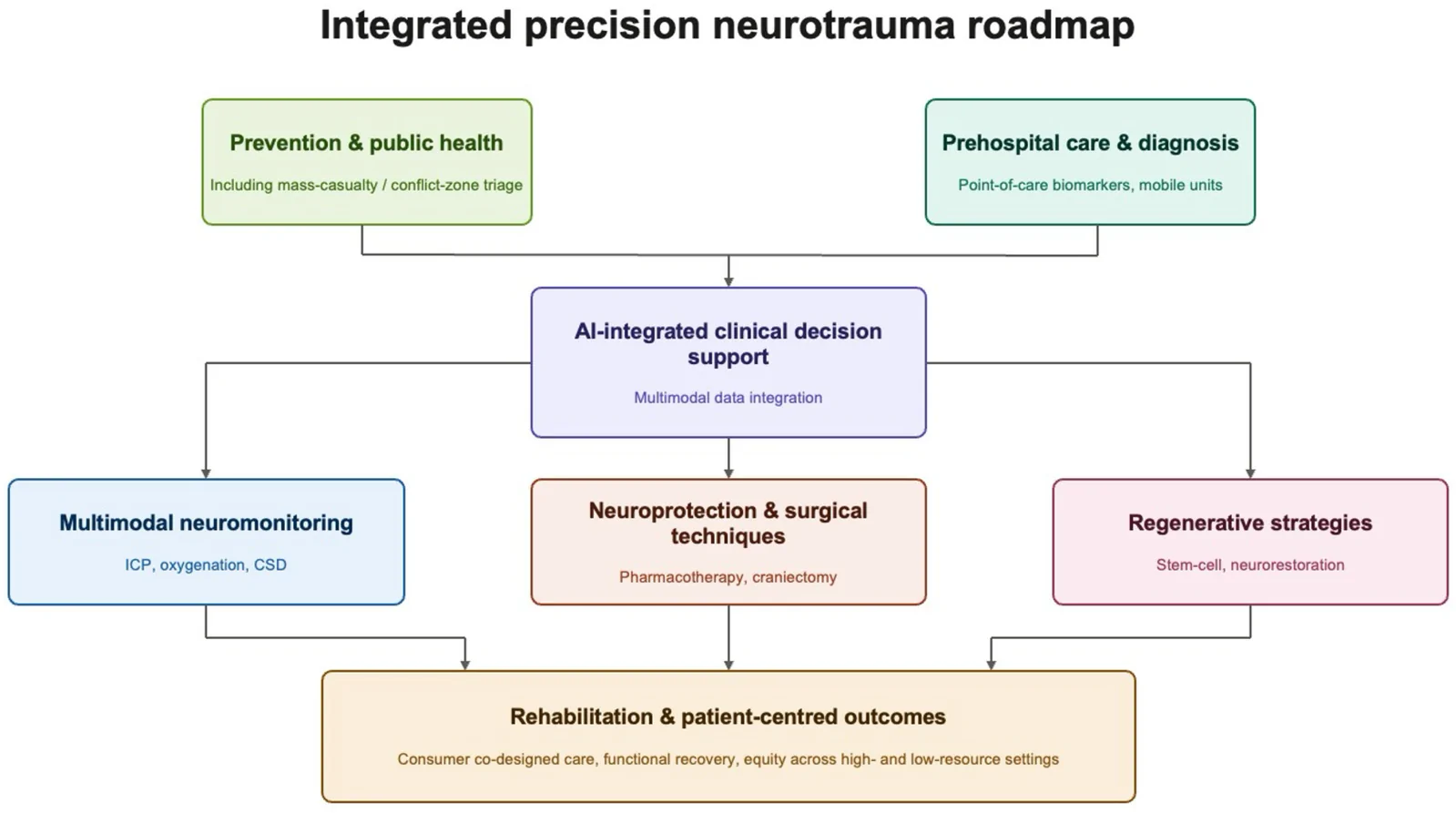
Integrated precision neurotrauma roadmap. Prevention and prehospital care feed into AI-integrated clinical decision support, which draws together multimodal neuromonitoring, neuroprotection, and surgical techniques. Regenerative strategies act in parallel; all domains converge on rehabilitation and long-term patient-centred outcomes. AI, artificial intelligence; ICP, intracranial pressure; CSD, cortical spreading depolarisation.

This narrative review examines emerging advances in traumatic brain injury classification, prehospital care paradigms, multimodal neuromonitoring, evolving surgical and medical therapies, rehabilitation and injury reversal strategies, and approaches to injury prevention. It has been deliberately framed as an expert-authored narrative roadmap and commentary rather than a systematic evidence synthesis; the relevant literature was identified through targeted Pub-Med/MEDLINE searches and supplemented by the authors’ clinical and research experience in neurotrauma, prioritising recent high-impact studies, established international guidelines, and seminal historical work, rather than a pre-specified systematic research protocol. The novelty of this manuscript lies in integrating these domains, together with contemporary and emerging interest in AI-supplemented decision-making, into a single, end-to-end precision neurotrauma model spanning injury prevention through to chronic rehabilitation and patient-centred outcomes (Figure 1; Table 1). Concepts that remain preclinical, early phase, or unvalidated are identified as such throughout.

## 2. Manuscript Body

### 2.1. Biological Reclassification of TBI

The understanding of the pathophysiology of TBI has evolved significantly from simple mechanical concepts that have their roots in ancient Egypt and the *Edwin Smith Papyrus* [5], and which persisted until the first half of the 20th Century with the adoption of the Monro–Kellie doctrine. The introduction of modern neuroimaging techniques, particularly computerised tomography (CT) and magnetic resonance imaging (MRI), and an explosion in neurocritical research which coincided with the clinical adoption of the GCS in the 1970s, resulted in the establishment of the “primary versus secondary injury” paradigm and the concept of the “golden hour” to limit injury by manipulating intracranial hypertension and avoiding cerebral hypoxia and hypoperfusion. Modern pathophysiology research is evolving well beyond simple structural or mechanical concepts to incorporate complex multi-system and temporal models including cascades of cellular and molecular inflammatory responses, electrochemical shifts, and potentially long-term metabolic abnormalities which may manifest in chronic traumatic encephalopathy (CTE), and the utilisation of biomarkers is increasing our understanding of the pathophysiology of concussion and repetitive injuries. Inflammatory cascades, excitatory neurotransmitter release, oxidative stress and chronic neurodegeneration are all being recognised as components of a multifaceted head injury pathophysiology phenotype, such that the primary versus secondary injury paradigm may in future recognise tertiary or even quaternary injuries [6]. Furthermore, interindividual genetic variation may influence outcome variability following TBI, and potentially as much as age, general health, and injury severity can, as identified in studies exploring the role of genetic effects on clinical outcome [7]. Beyond the influence of genetics on outcome, gene studies may reveal permanent changes in gene expression that could influence rehabilitation, as well as pathways to enable gene editing and expression which may limit the neuroinflammatory cascade [8].

The classification of disease remains vital in clinical medicine to produce accurate and standardised diagnoses, direct and prioritise treatment strategies, and to track public health. Current medical classification systems have their roots in botanical and animal taxonomy, and far from being an exception, TBI is in fact lagging far behind in the evolution of nomenclature and classification modernisation by its reliance on the GCS, which critics claim is far too basic to characterise head injury severity as simply mild, moderate or severe, and which indeed it was never intended to do. Although GCS remains useful in clinical assessment and monitoring, there is a continued push for the establishment and adoption of more encompassing head injury classification systems.

Advances aimed at addressing the limited assessment of brainstem integrity and injury heterogeneity inherent to the traditional GCS include the GCS-P [9] and FOUR score [10]. Beyond refinements of TBI classification based on neurological examination, there have been attempts to capture the implicit heuristics typically utilised in clinical application of the GCS, whereby the CENTER-TBI collaborators demonstrated emphasis on incorporating variables such as extracranial injury burden, mechanism of injury and radiological features in addition to GCS to subclassify TBI [11]. Building upon this concept, the recent NIH-NINDS supported framework including working groups led by Geoffrey Manley has proposed a multidimensional characterisation of TBI across four principal domains: ***C***linical (including GCS and pupillary reactivity), ***B***iomarker (blood-based measures), ***I***maging (pathoanatomical injury characteristics), and ***M***odifiers (patient and injury-related factors influencing presentation and outcome), with protein-based brain-enriched serum biomarkers in particular showing novel promise as a supplementary investigation for injury prediction, subclassification, and clinical prognostication. This framework has been suggested as a step toward a more biologically informed subclassification of TBI, particularly the addition of circulating biomarkers, and ultimately a focus towards more individualised therapeutic strategies [12,13,14,15]. Although still in need of validation, the CBI-M framework has demonstrated that applying additional pillars beyond a scoreable conscious state is conceptually important in the characterisation of TBI, which may then direct targeted therapies and improve overall clinical outcomes [16]. Without incorporating additional key physiological variables such as intracranial pressure (ICP) monitoring, critics of the CBI-M framework have indicated that its practical influence on acute critical care management remains limited, with no real-time application for treatment decision-making [17]; nevertheless, the expansion of a TBI classification system to capture modern and relevant variables sensitive to management and outcome is a much-needed evolution in clinical taxonomy. The disconnect between the simplicity of traditional GCS-based classification systems and the complexity of clinical TBI management is real; current management strategies incorporating GCS and radiological assessment guided by treatment guidelines (including the Brain Trauma Foundation, the American College of Surgeons and the SIBICC severe TBI algorithm) [18,19] do not capture the biological complexity and heterogeneity of TBI, and more research into modern classification systems such as CBI-M is warranted.

### 2.2. Time-Critical Care: Prehospital Triage, Imaging, and Haemostasis

R. Adams Cowley’s concept of the “golden hour” remains a foundational principle in modern trauma care [20]. In neurotrauma, timely intervention is arguably even more critical, as definitive diagnosis, localisation of injury, and access to multidisciplinary specialist expertise have traditionally been confined to tertiary neurosurgical centres. Consequently, a modified “scoop-and-run” approach that concurrently emphasises reduction in secondary brain injury by respiratory and haemodynamic stabilisation has dominated TBI retrieval systems [21]. Large geographic distances and difficult terrain in some global regions can prolong retrieval times, which can delay diagnosis and specialist interventions, and whilst the association between prehospital time and mortality in the modern era has been challenged [20,22,23], there is ongoing suggestion that neurological morbidity is influenced by prehospital time [24]. Furthermore, in the subgroup of patients with operative intracranial lesions, minimising time to neurosurgical intervention is difficult to ignore [25]. As a result, there is growing interest in technologies capable of augmenting prehospital management with the capacity to identify patients with operative pathology, provide basic haemostatic and metabolic resuscitation, and support basic neurosurgical interventions [26,27,28].

Among the most promising developments is the expanding use of mobile imaging platforms, which are becoming smaller, lighter, and less expensive with stepwise technological advances. Mobile CT stroke units have already demonstrated the feasibility of delivering advanced neuroimaging in the prehospital setting, although their application in traumatic brain injury remains comparatively limited [29]. Nevertheless, these systems offer the potential for earlier diagnosis of traumatic intracranial haemorrhage (TICH), more accurate triage, and expedited transfer to definitive neurosurgical care. Rational deployment of such units may additionally be guided through integration with emerging near-infrared spectroscopy (NIRS)-based technologies capable of detecting superficial or sizeable intracranial haemorrhage [30]. Earlier recognition of TICH can also assist selection of patients most likely to benefit from anti-fibrinolytic therapies [31].

Prehospital assessment may increasingly incorporate point-of-care coagulation platforms, enabling earlier identification of trauma-associated coagulopathy and initiation of targeted haemostatic therapies [32,33,34]. In severe TBI, where haemorrhage progression and secondary injury may critically influence outcome, reducing the interval between diagnosis, haemostatic correction, and operative intervention may represent a substantial advance in systems-based neurotrauma care. Ultra-early neurosurgical intervention is evolving with developments in powered cranial access devices, and electric auto-stop cranial drills and rapid access craniostomy kits may increase the plausibility of safe and early intracranial access in austere or prehospital environments and performed by general surgeons or healthcare professionals with limited specific neurosurgical training [35]. Collectively, these innovations raise the possibility that resuscitation, definitive diagnosis and initiation of emergent neurotrauma therapies may increasingly occur within the “golden hour,” progressively shifting advanced neurotrauma care ever closer to the point of injury.

### 2.3. Precision Neurocritical Care: ICP, Oxygenation, Metabolism, and Electrophysiology

Invasive ICP monitoring and ICP-guided neurocritical care remain well established in the management of severe TBI. Modern miniature piezoresistive strain-gauge microsensor transducers are now reliable and widely available and are considered standard of care. ICP monitoring remains inherently invasive and requires surgical insertion, and despite the theoretical uniformity of the intracranial contents, the recorded ICP measurements reflect only the local environment surrounding the implanted device [36]. ICP is also a surrogate physiological marker from which adequacy of cerebral perfusion and tissue oxygenation are inferred. Avoiding intracranial hypertension and limiting the ICP dose burden can improve mortality, but targeting isolated ICP thresholds has not consistently translated into dramatic improvements in neurological outcome; this was demonstrated in the BEST:TRIP clinical trial and argued in subsequent critiques of threshold-driven neurocritical care paradigms, but criticisms frequently neglect the finding that non-ICP-driven strategies are typically based on assumptions of intracranial hypertension [37]. The general acceptance of ICP monitoring has accelerated interest in alternative monitoring strategies directed more towards formal assessment of cerebral oxygen delivery and utilisation. Brain tissue oxygenation-guided therapy is currently being evaluated in several major international multicentre randomised trials including BOOST-3 and BONANZA, and cerebral oxygenation may ultimately become standard adjunctive monitoring alongside ICP [38,39]. Another evolving target of intracranial monitoring is cerebral compliance, which reflects the brain’s ability to accommodate intracranial volume changes relative to pressure variation, and may better represent intracranial dynamics than ICP measurements alone [40,41]. Gold-standard compliance measurement remains invasive and requires an infusion test to measure volume–pressure relationships and is still mostly under investigation, although other indirect and derived measurements such as the pressure reactivity indices (PRx) may provide indirect assessment utilising sophisticated analysis of relatively hidden data within the complex and high-frequency ICP waveform [42].

Oxygen delivery alone may not fully reflect cerebral metabolic function. A common final pathway across critical illness is impaired utilisation of delivered oxygen at the cellular level [43]; therefore, cerebral microdialysis offers a means of directly interrogating the cerebral interstitial environment and metabolic state, with measures such as the lactate–pyruvate ratio (LPR) demonstrating prognostic significance following severe TBI [44]. Despite this, the invasive nature, availability and technical complexity of microdialysis currently limits its use largely to specialised centres and for research applications [45,46,47]. With more widespread acceptance and with cost reduction associated with technological advances and economies of scale, it is feasible that multimodality monitoring via a single implantable device will replace ICP monitoring alone.

Distinct from traditional models of organ dysfunction centred upon oxygen delivery and consumption, the brain additionally possesses a uniquely electrophysiological dimension. Detection and therapeutic targeting of cortical spreading depolarisations (CSDs) has consequently emerged as another promising area of modern neurocritical care research [48]. CSDs represent propagating waves of neuronal and glial depolarisation associated with lesion expansion and poorer neurological outcomes following TBI [49]. Ketamine has attracted particular interest due to its NMDA receptor antagonism and apparent ability to suppress spreading depolarisations; early retrospective studies suggested ketamine sedation was associated with reduced CSD burden [50], and these findings were subsequently supported by prospective controlled crossover data demonstrating dose-dependent suppression of spreading depolarisations in severe TBI and aneurysmal subarachnoid haemorrhage [51]. These observations have subsequently led to ongoing prospective studies, including the INDICT trial [49] and KETA-BID study [52], evaluating electrophysiology-guided suppression of spreading depolarisations in acute brain injury.

Non-invasive methods for assessing intracranial dynamics are also emerging, although most remain investigational. Current approaches include optic nerve sheath diameter ultrasonography, pupillometry, transcranial Doppler ultrasonography, tympanic membrane displacement, and in neonates, the clinical assessment of fontanelle displacement. While these modalities may detect intracranial hypertension indirectly, none currently provide continuous physiological monitoring equivalent to invasive devices. Wireless implantable technologies using radiofrequency or Bluetooth transmission are likewise evolving and may eventually permit continuous intracranial monitoring with reduced procedural burden. Advances in microelectromechanical systems make the progression toward miniaturised wireless intracranial monitoring increasingly inevitable, and a reliable non-invasive or minimally invasive monitoring platform could substantially reduce the time to diagnosis of intracranial hypertension and potentially extend advanced neurotrauma monitoring into prehospital environments.

An important consideration in the development and implementation of emerging neurotrauma technologies is ensuring equitable performance across diverse patient populations and healthcare systems. External validation of near-infrared intracranial haemorrhage detection devices such as the Infrascanner in a Ugandan cohort demonstrated reduced diagnostic performance compared with earlier validation studies [53], highlighting the necessity for representative validation datasets and careful evaluation of bias during device development. Such findings reinforce that future advances in neurotrauma must not only pursue technological sophistication, but also equitable global applicability.

### 2.4. Therapeutic Evolution: Neuroprotection, Surgery, and Cranioplasty

The clinical translation of neuroprotective measures that have proven beneficial in the laboratory setting has to date overall been disappointing, and the fallacy of neuroprotective studies remains the expectation of specific agents to be effective for all TBI subtypes. Although sedation, paralysis, barbiturates and seizure prophylaxis are well established and extensively utilised to minimise secondary injury, none of these agents is technically directly neuroprotective. The two main targets of clinical neuroprotection in early research have been hypothermia and corticosteroid therapy, but both have been disproven in clinical trials with adverse effects demonstrated to outweigh any benefits, and the use of high-dose steroid therapy is specifically not recommended in TBI based on Class I evidence [54]. One of the issues that may be restricting uptake of cooling and/or steroid therapy is the ability to identify certain individuals who may yet benefit from these treatments, such that a subset of patients with TBI may in fact have improved clinical outcomes with the application of neuroprotection, whereas others may not, and steroids in particular may have a role in physiological rather than supratherapeutic doses in settings of deficiency. Given the heterogeneity of the pathology and the population, it is entirely feasible that certain individuals may in fact do better with laboratory recognised but clinically disproven neuroprotective techniques; however, extensive high-quality research, including translational studies and randomised clinical trials based on the existing evidence and animal models, will likely be required to identify appropriate future usage and the efficacy of neuroprotective measures [55].

The current understanding of the neuroinflammatory cascade following TBI is evolving and revolves around research into excitotoxicity, oxidative stress reactivity and neurodegenerative pathways. Cerebral oedema following TBI remains a significant global challenge, and no single drug agent has yet been proven to limit post-traumatic swelling and/or interrupt the inflammatory cascade. Novel agents are continually being investigated based on increasing knowledge of the pathophysiology of changes within the central nervous system milieu following injury, and several have shown promise in pre-clinical trials. Such agents include the Transient Receptor Potential Canonical (TRPC) ion channel blocker NYR-BI03 (Xolatryp™, Nyrada Inc., Sydney, Australia) which acts by reducing calcium overload and limiting cerebral ischaemia, synthetic peptides (including Cerebrolysin and Trofinetide) which may limit inflammation and apoptosis, natural anti-oxidants such as magnesium, Omega-3 fatty acids and vitamin C, and Substance P and NK1 receptor antagonists which can limit neuroinflammation and abnormal blood–brain barrier permeability to reduce post-traumatic vasogenic cerebral oedema. Advances in the understanding of traumatic neuroinflammation may inform the development of targeted drug therapies, and as more agents and pathways are identified, neuroprotection strategies could evolve towards a multi-targeted pharmacological approach aiming to limit or accelerate resolution of cerebral oedema and the secondary mechanical effects of intracranial hypertension, although this remains an area of active preclinical and early phase clinical investigation.

The trauma craniotomy has its roots in the very beginning of the 20th Century by Swiss surgeon Theodor Kocher [56]. Further refinement of the technique developed in association with major conflicts in the following 70 years and particularly from observations made by Harvey Cushing in the First World War, but also by trauma surgeons in World War II, the Korean War and the Vietnam War. A bifrontal craniectomy was commonplace originally and particularly prior to the advent of modern neuroimaging; however, the unilateral fronto-temporo-parietal haemicraniotomy (or haemicraniectomy) is now the most common operation performed, and the technique has been mostly unchanged in the last 50 years. Decompressive craniectomy (DC) has been moderately controversial in recent times and has been the subject of several large randomised controlled trials, but the current consensus within the community is that DC has its place following TBI in limiting intracranial hypertension and reducing mortality rates, but at the cost of an increased number of clinically dependent survivors. As already stated, ML algorithms may help identify patients who would benefit from specific surgical interventions, and the decision to offer a patient DC could be derived based on clinical, radiological and physiological data compared with historical datasets to limit mortality and maximise favourable and patient-specific acceptable neurological outcomes.

Refinements to the unilateral-DC technique continue to evolve, with adoption of alternative incision patterns designed to address limitations of the traditional ‘reverse question mark’ or ‘Dandy-incision’ to improve posterior exposure [57,58], maximise craniectomy size [59], and limit wound complications [60]. The optimum size of DC remains an area of debate, and whilst a minimum area of 12 × 15 cm [61,62] in conjunction with craniectomy extension to the middle fossa floor specifically for the management of uncal herniation is the current recommendation, many neurosurgeons will perform a DC larger than these dimensions if anatomically possible to minimise parenchymal injury associated with iatrogenic transcranial herniation and to limit venous obstruction.

Despite the understanding that DC size is important, the adherence to this recommendation is poor [63], and this may at least partly relate to historical uncertainty. In the RESCUE-ASDH study [64], disability and quality-of-life outcomes following surgical evacuation of traumatic acute subdural haematomas were found to be comparable in individuals undergoing craniotomy with bone flap replacement versus DC in cases of clinical equipoise. One result of the publication of this landmark study may be a shift towards larger planned surgical decompressions and fewer incidental DCs arising from otherwise solely evacuative procedures. Irrespective of this, evidence supporting superior neurological outcomes with increasingly larger craniectomy size remains limited, and further studies are required [63,65].

The intuitive rationale underpinning decompressive strategies has led to further therapies aimed at improving accommodation of cerebral swelling but reducing the incidence of complications typically associated with DC. The hinge craniotomy (HC) has been proposed with numerous variations, all of which fundamentally describe a craniotomy, with disconnection of a bone flap followed by partial fixation where the replaced bone remains mobile, allowing and accommodating for pressure effects and cerebral swelling, and theoretically obviating the need for subsequent reconstructive cranioplasty [66,67,68,69,70]. Broad adoption of the HC remains limited despite evolving evidence [71], and ongoing concerns exist regarding their effectiveness in ICP control compared with formal DC such that HC should probably primarily be evaluated as an alternative to conventional craniotomy for TICH evacuation where the management of modest perioperative swelling is less well understood.

As per the Monro–Kellie doctrine, the volume of the intracranial contents can be additionally augmented by reducing the amount of ventricular cerebrospinal fluid (CSF). In this context, the use of external ventricular drains (EVDs) is conceptually attractive, serving both as a monitor of intracranial pressure and as a therapeutic means of CSF diversion. However, whether this dual function translates into superior clinical outcomes compared with intraparenchymal monitoring remains uncertain, and large adjusted observational studies have produced conflicting results [72,73], whilst a randomised comparison (despite being confusingly labelled as observational) reported favourable outcomes with EVD-based monitoring [74]. Additional studies comparing ICP monitor-driven therapy with EVD pressure monitoring and CSF drainage management are therefore valid and would provide useful information regarding the utility of EVD insertion as standard of care in TBI management. EVD insertion can be anatomically and technically challenging in TBI when ventricular calibre is small, although the application of neuronavigation and stereotaxy may increase success rates, and CSF drainage via surgical cisternostomy has been proposed as an alternative. Studies promoting this technique have been criticised for their methodological rigour and potential over-enthusiastic conclusions [75], but a systematic review of the existing literature has found that cisternostomy when performed with DC may have an association with lower mortality and a reduction in mechanical ventilation [76].

Other subspecialities within neurosurgery have undergone substantial evolution through the adoption of endovascular therapies, and these technologies may have an emerging role in TBI management. Endovascular intervention in blunt cerebrovascular injury is well established, and the experiences in venous sinus stenting in IIH [77] have prompted consideration of whether modulation of cerebral venous outflow could have future applications in TBI [78]. Translation of these concepts into neurotrauma would require physiological validation, and would also need to incorporate the not insignificant intracranial haemorrhage risks associated with antithrombotic therapy following acute TBI. Chronic subdural haematoma intervention has had a recent renaissance, particularly regarding endovascular techniques for middle meningeal artery embolisation (MMAE) to reduce recurrence rates, and these interventions and strategies will continue to evolve along with other endovascular interventions for intracranial vascular pathology, particularly cerebral aneurysms. MMAE has proven to be beneficial clinically but is still to be extensively investigated with randomised clinical trials, and significant financial limitations remain secondary to the relatively high cost of endovascular coils and particulates. Endovascular techniques are unlikely to be adopted yet in low- and middle-income countries (LMICs), but the effect of meningeal arterial occlusion could be replicated at the time of burr hole surgery relatively easily by electrical coagulation, and this simple technique will be the subject of ongoing investigation.

Reconstructive cranioplasty is well established for not only safety and cosmetic purposes but also to improve rehabilitation prospects following DC, and the evidence that cranioplasty performed early after DC rather than later to improve neurological outcome is becoming increasingly clear [79]. An optimal window for cranioplasty probably exists for individual patients during which the benefits of reconstruction outweigh the surgical risks, and recognising and taking advantage of this window should become a routine part of TBI management. Bone flap storage research will need to address ongoing issues relating to autologous resorption, and synthetic flaps (whether titanium, acrylic or PEEK) will be increasingly 3D printed, which will reduce production costs and will increase availability as 3D printers become more commonplace. Sonolucent cranioplasty plates are currently being evaluated as a means of facilitating postoperative ultrasound surveillance, and these could potentially provide rapid bedside diagnostic assessment in the postoperative setting [80].

### 2.5. Artificial Intelligence and Computational Decision Support

It is plausible that AI will become an increasingly routine adjunct to clinical management across many disease processes affecting the human body and TBI is unlikely to be an exception to this; however, this will require extensive preclinical validation prior to the routine adoption of AI-integrated support in clinical TBI decision-making [81]. The explosion of AI in recent times has been nothing short of phenomenal, and the ability of machine-learned algorithms to analyse great amounts of data at incredible speed fits exceptionally well into the future clinical management of TBI. At present, AI seems best utilised for prognostication and outcome prediction modelling [82], but also has applications in diagnosis, neuroimaging, and rehabilitation [83]. Current large dataset models such as CRASH [84] and IMPACT [85] provide predictive values for death and disability based on a limited number of clinical variables using multivariable logistic regression, and these models have been validated but are still not entirely accurate and may incorrectly prognose up to one in four patients [86].

Critical in the success of artificial modelling is the ability of computers to identify patterns and rules in data but without being specifically programmed; this subfield of AI is better defined as machine learning (ML). Common ML classification algorithms include Random Forest (RF) and Support Vector Machine (SVM), and ML algorithms have been demonstrated to outperform traditional statistical-based models in predicting favourable clinical outcome and mortality following TBI, with in-hospital mortality prediction accuracy of up to 95.6% [87]. As ML algorithms become more sophisticated and as increasing swathes of data become available, the accuracy of these models will increase, and the prognostication of TBI will become automated and potentially provide highly useful information extremely early in the disease process to guide critical treatment decisions, escalate or pre-empt escalation of interventions, and even discontinue therapy in the event of an expected poor outcome.

Radiological diagnosis of TBI and particularly the presence of TICH remains critically important to guide prognostication and clinical decision-making. Early recognition of TICH patterns results in faster times to therapeutic intervention to ultimately improve clinical outcomes, and the digitisation of neuroimaging with associated data extraction from DICOM files suits the application of ML algorithms particularly well. Several ML algorithms have been investigated for speed and accuracy, and recent studies have demonstrated identification of the presence of TICH within 2 min of scan completion with 85% sensitivity and 98% specificity [88]. Application of ML algorithms to CT analysis following TBI will facilitate standardisation of diagnosis, which will, in turn, assist the development and adoption of currently proposed classification systems like CBI-M, as will the incorporation of serum biomarkers and longitudinal profiling as this emerging field becomes increasingly routine [13]. Identification of other critical intracranial radiological findings beyond TICH, such as skull fractures, midline shift or diffuse cerebral oedema (as a surrogate for intracranial hypertension), will be beneficial in facilitating earlier interventions, and early reporting of cerebral imaging, particularly in non-trauma centres, may also result in faster retrieval and benefit those in rural and remote settings [89].

It is necessary to remain mindful that the clinical utility of AI in neurotrauma is unlikely yet to reside in reproducing predictions that clinicians already make with confidence. Accordingly, impressive discrimination metrics such as high area-under-the-curve (AUC) values should be interpreted cautiously, as these may largely reflect accurate prediction of clinically obvious outcomes rather than any meaningful augmentation of complex clinical decision-making, and the comparative utility of ML algorithms utilising low-granularity data has demonstrated no benefit when compared with regression modelling [90]. The advantage of AI algorithms likely lies in their capacity to integrate high-dimensional, multimodal, and temporally granular physiological data streams that exceed human cognitive capacity [91,92]. While traditional human-patient counselling is restrained by discussion of aggregate treatment effects derived from randomised controlled trials, such approaches inadequately account for the highly individualised and temporally evolving nature of neurotrauma physiology. By contrast, AI-assisted systems may integrate continuously evolving multimodal data to generate personalised probabilistic estimates of treatment response. Ultimately, this may bring us closer a highly individualised therapeutic rationalisation where clinicians can identify and provide the right treatment, for the right patient, and at the right time.

Future evaluation frameworks should therefore focus on whether AI meaningfully improves decision quality in ambiguous cases, allows identification of overlooked signals from highly granular data, or supports personalised recommendations by assessing historical data for an individual patient. Evaluation of this may involve retrospective analyses of highly granular multimodal neurocritical care datasets, whereby expert clinician groups are provided with physiological, radiological, laboratory, and monitoring data and asked to generate prognostic estimates or management recommendations. These clinician-derived assessments could then be directly compared with AI-generated predictions or recommendations derived from the same datasets, permitting evaluation of whether AI could augment current standards of care. Such a shift may prove more clinically meaningful than isolated comparisons of prognostic discrimination alone, which currently dominate much of the neurotrauma AI literature [93].

Although neurotrauma datasets have progressed substantially from earlier prognostic cohorts such as CRASH and IMPACT to modern precision-neurotrauma initiatives including TRACK-TBI and CENTER-TBI, acquisition of highly granular multimodal monitoring data remains restricted to a limited number of centres. As such, the potential of AI-assisted neurocritical care may currently be constrained less by capability than by the resolution and representativeness of available datasets. Overall, the integration of AI into clinical neurosurgery remains in its relative infancy and is accompanied by substantial ethical, practical, and regulatory challenges, including clinician and patient acceptance, algorithmic bias arising from training datasets, safety oversight, explainability, and medico-legal accountability when AI-informed recommendations contribute to adverse outcomes. Intensive multicentre collaborative international efforts will likely be required to define robust methodological frameworks to develop these highly granular multimodal datasets, which are necessary for clinically meaningful evaluation and the implementation of AI-assisted neurotrauma systems.

### 2.6. Chronic TBI, Rehabilitation, and Patient-Centred Outcomes

Rather than a one-time event, TBI is being increasingly recognised as a chronic health condition with long-lasting cognitive, physical and emotional impairments [94,95,96]. As such, TBI must be considered a dynamic rather than a static phenomenon, and management strategies must transition from the initial acute phase and well beyond into longer-term rehabilitation and reintegration into the community. Recent interest in concussion injuries, particularly in sports and the military, and ongoing research into the long-term effects of repetitive injuries and chronic traumatic encephalopathy (CTE) are excellent examples of the shift towards understanding TBI as a chronic health condition, and studies exploring longitudinal serum biomarker analysis will likely strengthen this narrative [97]. Repetitive head strikes have long been recognised to have an association with dementing illnesses in “punch drunk” pugilists, but earlier this century, the effects of repetitive injury became increasingly investigated and particularly driven by interest in cognitive and psychological changes observed within the community of American football players. Post-mortem studies have demonstrated aggregation of hyperphosphorylated tau (p-tau) as the pathognomonic lesion, which may or may not be cortical sparing [98]. The neurocognitive effects of CTE have similarities with other age-related p-tau associated neurodegenerative diseases, particularly Alzheimer’s Disease (AD), and an understanding of pathological processes will have a significant overlap in the treatment of not only chronic TBI conditions but age-related neuropathology [99].

Psychostimulants have been used following TBI, particularly for issues relating to arousal, wakefulness and fatigue, with common agents including methylphenidate, amantadine and dextroamphetamine. Beyond alertness, cognitive processing and behaviour regulation may improve with central nervous system (CNS) stimulation, and although commonly prescribed, the use of stimulant drugs is generally ad hoc with limited well-designed large-scale clinical evidence [100]. Newer CNS drugs are under investigation, and the non-amphetamine psychostimulants modafinil and armodafinil have demonstrated potential in improving wakefulness following TBI but still require extensive clinical trials to prove clinical efficacy [101]. An evolving pre-clinical concept is that of environmental enrichment where motor, sensory, and social stimulation has been demonstrated to enhance recovery post acquired brain injury [102], and application of this strategy may be associated with enhanced functional recovery [103]. Nevertheless, adoption of environmental enrichment techniques is limited by lack of human data, issues relating to clinical translation, and overall expense.

Current technology has the potential to significantly modify traditional approaches to TBI. Telehealth has the ability to provide access to services otherwise restricted by geography; AI therapists in combination with virtual reality [104] could reproduce rehabilitation environments that would otherwise require inpatient admission in specialist centres, and artificial speech and language therapy via increasingly sophisticated linguistic models have already demonstrated promise in post-stroke dysphasia [105]. Patient behaviour and clinical indicators such as radiological findings or serum biomarkers may be repeatedly analysed by ML algorithms, and subtle changes in an individual may indicate a dynamic shift in their chronic TBI, which may, in turn, prompt medical, physical, radiological and/or psychological intervention in response to these changes [83].

Also evolving is the field of non-invasive neurostimulation as an adjunct to the rehabilitative process [106]. In parallel, invasive neuromodulation techniques, albeit still exploratory, continue to undergo refinement [107]. Advances in robotics and exoskeleton devices have the ability to restore physical disabilities, and robot-assisted gait training devices have been demonstrated to improve functional improvement measurement scores in patients with acquired brain injury [108].

Of critical importance in delivering holistic rehabilitation services is the requirement for coordinated multidisciplinary input [109]. As neurorehabilitation increasingly evolves toward individualised and precision-based paradigms, integration of diverse clinical expertise alongside advanced neurophysiological, imaging, and functional assessment modalities may become increasingly important in tailoring rehabilitation trajectories to individual patient needs. Finally, current neurological function and clinical outcome scales are in need of revision to remain relevant and reflective of real-world standards. Although the Glasgow Outcome Scale-Extended (GOS-E) is still the global measure for clinical outcome in TBI and other neurological trials, it is a crude measurement of functional status and frequently unfairly dichotomises into a favourable or unfavourable outcome without acknowledging acceptable levels of dependency [110]. Unlike GCS, which remains a simple but highly relevant instrument in clinical TBI assessment and which has adapted beyond its intended use and as such has aged remarkably well, the recognised limitations of GOS-E require more sophisticated alternatives such as the proposed Functional Status Examination, which may better incorporate the impact of TBI on social and psychological measures and which may arguably be a more accurate determination of acceptable disability [111,112].

### 2.7. Prevention and Global Equity

A comprehensive understanding of TBI management must incorporate injury prevention strategies. Patterns of TBI vary geographically; in high-income countries (HICs), falls from standing height among older adults are an increasingly important contributor, whereas road traffic collisions remain a predominant mechanism in most LMICs [113]. Accordingly, prevention strategies must adopt geographically and socio-culturally informed approaches targeting at-risk populations through interventions developed in partnership with local stakeholders and communities; initiatives that promote safety awareness regarding falls in the elderly will have a larger impact in HICs, and efforts into mandatory helmet legislation will have a larger positive effect in road traffic mortality and morbidity in African nations [114,115]. Attention should also be directed toward patient populations that may be underrepresented in traditional guideline frameworks, including individuals experiencing domestic violence, homelessness, mental and/or physical disability, or social marginalisation, as well as Indigenous populations whose needs may differ substantially across healthcare systems. In parallel, increasing awareness of sports-related concussion has prompted greater regulatory oversight and evolving return-to-play frameworks within professional and recreational sporting organisations. Nevertheless, improving accessibility of concussion recognition and management guidelines for community and amateur athletes remains an important ongoing priority.

Mass casualty events, including natural disasters (earthquakes, tsunamis and avalanches) and armed conflict, present distinct challenges for TBI triage and management that differ substantially from routine civilian trauma systems and frequently require a different approach. Recent wartime experiences, including from contemporary conflict zones, highlight considerations specifically relevant to TBI, including safety and logistical concerns, prolonged prehospital transport times, damage control neurosurgical interventions frequently performed within resource-constrained and unstable conditions, and the management of penetrating ballistic and blast-related head and facial injuries which differ significantly from closed-head injuries that dominate the civilian TBI literature. These experiences have prompted renewed interest in simplified, resource-appropriate triage protocols and surgical techniques that can be rapidly and consistently deployed, potentially by non-specialist teams prior to definitive transfer, with lessons that are increasingly informing civilian mass-casualty and disaster preparedness planning in both HICs and under-resourced settings.

Established evidence-based medicine (EBM) clinical guidelines for the management of TBI include those developed by the Brain Trauma Foundation [62] and the SIBICC algorithm [19]. Although such recommendations are not mandatory, they provide an important framework for EBM management and have been associated with reduced TBI-related mortality [116], and efforts to evaluate adherence to these guidelines should ideally occur in an iterative and cyclical manner. These guidelines may have less application in bedside practice and particularly in less resourced environments; therefore, targeted efforts are required to ensure there is adaptation of recommendations for diverse clinical environments, such as the CREVICE protocol providing recommendations where access to modalities such as intracranial pressure monitoring is limited [117].

A natural limitation of current established guidelines is the inherent need for clinical evidence to support the provision of recommendations. When evidence is lacking, clinical recommendations are less robust and therefore should be combined with the release of high-quality expert consensus statements to address gaps identified in the literature. Guideline development is often time-intensive such that recommendations may lag significantly behind rapidly evolving evidence and this is becoming increasingly obvious in the digital age. Supervised AI systems may have a future role in supporting more rapid and iterative updates to recommendations, and dynamic guidelines supported by AI remain alternatives to enhance the contemporaneity of clinical recommendations.

### 2.8. Regenerative and Restorative Strategies

Once a human brain has been damaged, the resulting tissue loss and/or neuronal disconnection is currently irreversible and is the basis for the concept of a primary brain injury. Advances in organic tissue reconstruction have enabled regeneration of comparatively simple structures such as ears, noses, or digits, but the human brain’s complexity, with close to 100 billion neurons and up to 1 quadrillion synaptic connections, means that meaningful neuroregeneration remains a substantially more distant and uncertain goal, achievable only with considerably further technological and biological advances.

It is now accepted that the mammalian CNS has some regenerative capabilities via endogenous neural stem cells (NSCs) that are located in the subventricular zone (SVZ), and these can migrate and differentiate into functional neurons [118,119]. CNS repair mechanisms utilising NSCs therefore have attracted interest to reestablish neuronal pathways lost via injury and to remodel circuits to restore lost function. Rodent studies in the 1990s [120] demonstrated improved motor and cognitive function in TBI following the implantation of foetal mouse cortex, and further studies have demonstrated regeneration of spinal cord pathways following implantation of embryonic stem cells (ESCs) [121]. Current research recognises four major stem cell types of interest: mesenchymal stem cells (MSCs), induced pluripotent stem cells (iPSCs), ESCs, and umbilical cord blood stem cells, each with their distinctive advantages and disadvantages when applied to TBI. ESCs are totipotent cells and can differentiate into any cell type but are derived from living embryos and have significant ethical considerations; MSCs, on the other hand, are multipotent but can be derived from mesoderm and have been isolated from multiple available sources such as bone marrow, adipose tissue, and placenta, and ethical concerns are therefore considerably less. The mechanisms of stem cell therapy following TBI depend highly on the time of implantation relative to the injury, such that early benefits are associated with limiting the spread of the injury, with later recognised benefits transitioning towards angiogenesis, repair of the extracellular matrix, and neural remodelling. Implanted stem cells migrate towards the site of injury via chemokines, cytokines and signalling pathways, and they also secrete bioactive exosomes which act as important information carriers and promote cellular repair. Some studies have investigated the role of exosomes without stem cell implantation, with benefits associated with reduced immunogenicity and easier administration given that they readily cross the blood–brain barrier (BBB) and can also therefore be easily utilised for CNS drug delivery.

The clinical application of stem cell and/or exosome therapy in TBI remains complex; the diffuse and heterogeneous nature of TBI makes targeting therapy delivery challenging when compared with more discrete injuries such as stroke or spinal cord injury, although advanced imaging may help identify focal regions of tissue injury suitable for direct implantation. Intravenous administration remains attractive because of its relative simplicity and suitability for agents such as exosomes that have BBB permeability, but therapeutic efficiency may be limited by pulmonary sequestration and reduced lesion engraftment [122]. Intrathecal delivery is conceptually appealing for more diffuse CNS distribution; however, it remains unclear whether observed benefits reflect true cellular engraftment or predominantly paracrine effects [123,124]. Recent clinical trials have demonstrated promise for stem-cell therapy delivered through direct cerebral implantation [125,126], although important questions remain regarding real-world efficacy, patient-level benefit, and long-term safety. Autologous adipose tissue-derived MSCs may represent a potentially acceptable and scalable cell source, but comparative efficacy across cell types and delivery routes remains uncertain. Ethical considerations are also likely to differ according to cell source, particularly when considering cord-blood-derived products or more developmentally immature cell lines. Given the vast diversity in existing study design and outcome measures of existing TBI-related stem-cell research [119], consensus-based frameworks that standardise outcome and promote inter-laboratory collaboration may in future allow integration of distributed expertise to support future robust pooled analyses.

## 3. Discussion

The management of TBI has evolved significantly since the beginning of the scientific revolution, and continuous scientific investigation supplemented by clinical research will fuel the ongoing evolution of the discipline interspersed with anomalous leaps of revolution. Technological advances in recent times have been nothing short of phenomenal and human ingenuity cannot be underestimated: the Wright brothers first flew in December 1903, only 69 days after the *New York Times* had declared that powered flight would require the continuous and combined mathematical evolution of up to “ten million years”, and, within another 66 years, Neil Armstrong had walked on the moon having been delivered there by NASA’s Apollo 11 spacecraft launched by the powerful Saturn V rocket. Similarly, multifaceted advances in managing TBI have evolved with modern technology, driven by tireless clinicians and researchers in desperate desire to improve clinical outcomes in what is frequently a devastating injury with profound impacts on the community, and on a global scale.

TBI management is overdue for a paradigm shift, and the evolving understanding of injury pathophysiology will continue to challenge previously accepted beliefs. Landmark examples of knowledge shift include the DECRA Trial from 2011 [127] and the subsequent RESCUEicp Trial published five years later, which challenged and then reinforced the concept that aggressive reduction in intracranial hypertension via surgical decompression would result in improved clinical outcome; surgery improves survival but at the cost of neurological disability initially at least; however, longer term data from RESCUEicp suggest that a third of survivors are dependent outside of home, with another third dependent within their home by 24 months [128,129]. Results from these studies have fundamentally reshaped modern neurotrauma, shifting emphasis away from ICP as an isolated therapeutic target towards a broader consideration of cerebral physiology, quality of survival, and individual patient trajectories towards a chronic, dynamic, and evolving clinical state. This shift has accelerated interest in precision physiological management strategies, including optimisation of cerebral perfusion pressure (CPPopt) through autoregulation-guided therapy [130,131], alongside incorporation of multimodal neuromonitoring integrating ICP, cerebral oxygenation, electrophysiology, cerebral metabolism, cerebrovascular reactivity and long-term management strategies. Furthermore, the recognition and acceptance that TBI is not a singular disease entity but rather a heterogeneous spectrum of injuries and chronic illness with differing biological responses and recovery trajectories should deliver increasingly patient-specific therapies and individualised care, and this will hopefully translate into improved functional outcomes.

The future of neurotrauma may therefore depend upon increasingly sophisticated subclassification of TBI through integration of multimodal physiological data, advanced imaging, biomarkers, genomics, and computational modelling. Bayesian and ML approaches may eventually enable increasingly accurate prediction of outcome trajectories, facilitate more informed clinical discussions, and allow truly personalised therapeutic strategies directed toward maximising patient-specific meaningful neurological recovery. Beyond this, regenerative approaches including neurorestoration, stem-cell therapies, and bioengineered reconstruction may ultimately redefine what is considered recoverable after a severe brain injury. While many of these possibilities currently appear aspirational, the history of neurotrauma repeatedly demonstrates that transformative advances often become self-evident only in retrospect.

Despite all of this, as technology accelerates toward these ambitions, it is essential to remember the human cost associated with the clinical advancements thus far achieved, the countless lives lost, the families and communities destroyed, and the huge global economic burden. Progress in neurotrauma research has been built upon generations of diverse patients, communities, clinicians and scientists driven by a willingness to dedicate themselves to understanding and improving the care of patients with brain injury. Ultimately, the value of any innovation must be measured not only by its technological sophistication, but by the extent to which its benefits can be delivered equitably, globally, and in service of preserving individually significant meaningful human function. Achieving this goal extends beyond the neurocritical care unit and requires continued collaboration between clinicians, scientists, healthcare systems, policymakers, advocacy organisations, and patients themselves. Maintaining this collective momentum remains essential if the aspiration of precise, accessible, and meaningful neurotrauma care is to become reality.

## 4. Integrated Future Roadmap

Future directions in neurotrauma are likely to be shaped by increasing movements towards precision-based, data-driven, and patient-centred models of care spanning the prehospital environment through to long-term neurorehabilitation. Efforts should therefore be directed toward developing and validating modernised, TBI-specific retrieval paradigms that incorporate portable, hand-held diagnostic technologies for the early detection of operable traumatic intracranial haemorrhage. Where high-risk lesions are identified, this could trigger deployment of specialised mobile neurotrauma units equipped with clinical expertise, CT imaging capability, and the capacity to address common reversible contributors to secondary injury, including coagulopathy and electrolyte disturbance. In selected cases, the ability to deliver time-critical stabilisation or temporising surgical intervention before definitive transfer may reduce secondary brain injury while minimising delays to specialist care.

Collaborative efforts to adopt and develop the CBI-M framework are another priority, and offer an opportunity to support precise patient stratification and targeted therapy selection.

AI should be leveraged as a clinician decision-support tool, particularly where it can integrate real-time multimodal monitoring data at a scale and temporal resolution beyond unaided human cognition. Rather than replacing clinicians, AI may be most valuable to begin with when used to identify clinically relevant signals from complex physiological, radiological, laboratory, and treatment-response data streams, thereby supporting dynamic and individualised decision-making.

Continued research is required to optimise surgical techniques, enhance neurorehabilitation, and explore regenerative therapies such as stem-cell-based interventions. These efforts should occur within coordinated methodological frameworks or consensus-guided structures to standardise outcomes, improve reproducibility, and enable individual patient data meta-analyses. Such approaches may help convert distributed research efforts into an integrated evidence base for the overall benefit of patients.

Future management guidelines should better address the needs of marginalised populations and include pragmatic recommendations for low- and middle-income countries, where resource constraints may substantially alter implementation.

Finally, incorporation of patient lived experience and consumer engagement into intervention design and outcome selection may facilitate treatment paradigms that more meaningfully reflect the priorities, functional goals, and quality-of-life concerns of diverse neurotrauma survivors.

## 5. Conclusions

Precise, accessible, and meaningful neurotrauma care will likely be advanced by more granular definitions of TBI and by harnessing AI to identify clinically relevant signals from multimodal monitoring data. Over the next 50 years, these approaches may underpin progress toward individualised therapeutic targeting and support consumer–physician co-designed models of care that reflect priorities of those with lived experience with TBI.

## Figures and Tables

**Table 1:** Integrated precision neurotrauma roadmap, summarising the current state, emerging tools, evidence level, implementation barriers, and research priorities across major domains of TBI management.

Domain	Current State	Emerging Tools	Evidence Level	Implementation Barriers	Research Priority
Prehospital care & diagnosis	GCS-based clinical triage; imaging-dependent diagnosis upon hospital arrival	Point-of-care biomarker assays; handheld ultrasound/CT; mobile neurotrauma units	Early clinical/feasibility	Cost; device portability; prehospital workforce training; regulatory approval	Prospective validation of point-of-care diagnostics against neuroimaging
Multimodal neuromonitoring	ICP and cerebral perfusion pressure monitoring in specialised centres	Brain tissue oxygenation, microdialysis, cortical spreading depolarisation monitoring, autoregulation-guided CPPopt targets	Established (ICP) to early clinical (multimodal integration)	Invasiveness; cost; need for specialist interpretation; limited availability outside of tertiary centres	Prospective trials of multimodal vs standard ICP and CPP- driven care
Neuroprotection & pharmacotherapy	Supportive/symptomatic management; no proven pharmacological neuroprotectant	Anti-inflammatory/anti-oxidant agents; blood–brain barrier stabilisers; multi-target pharmacotherapy	Preclinical to early phase clinical	Prior neuroprotectant trial failures; injury heterogeneity; narrow or variable therapeutic windows	Biologically stratified trial design linked to CBI-M classification
Surgical techniques	Decompressive craniectomy; established cranioplasty timing principles	Minimally invasive/keyhole approaches; 3D-printed synthetic flaps; individualised cranioplasty timing	Established to early clinical	Surgeon training; cost and availability of custom implants; variable institutional resourcing	Long-term functional and cosmetic outcome data for novel techniques
AI & computational decision support	Prognostic models (e.g., CRASH, IMPACT); limited clinical AI deployment	Real-time multimodal data integration; AI-supported dynamic guideline updating	Early phase/largely retrospective	Data standardisation; explainability; clinician trust; regulatory pathways; liability	Prospective validation of AI decision-support in real-world neurocritical care
Rehabilitation & long-term outcomes	Multidisciplinary rehabilitation; standard disability outcome measures (e.g., GOSE)	Digital/telehealth therapies; AI-assisted speech/language rehabilitation; refined disability measures (e.g., Functional Status Examination)	Early clinical	Access and equity; funding models; outcome-measure standardisation	Consumer co-designed rehabilitation outcome frameworks
Regenerative strategies	No proven regenerative therapy for restoring lost neuronal tissue	Stem-cell-based interventions; neurorestorative approaches	Preclinical	CNS regenerative complexity; safety; delivery methods; translational uncertainty	Standardised preclinical frameworks enabling pooled/meta-analytic evaluation
Prevention, public health & guidelines	Established BTF/SIBICC guidelines; region-specific prevention initiatives	Context-adapted protocols (e.g., CREVICE) for resource-limited settings; mass-casualty and conflict-zone triage frameworks	Established (guidelines) to emerging (adapted protocols)	Guidelines lag behind evidence; HICs/LMICs resource disparity; applicability in mass-casualty/conflict settings	Pragmatic LMICs and conflict/mass-casualty recommendations; AI-assisted iterative guideline updating

## Data Availability

No new data were created or analyzed in this study. Data sharing is not applicable.
